# Interacting effects of early dietary conditions and reproductive effort on the oxidative costs of reproduction

**DOI:** 10.7717/peerj.3094

**Published:** 2017-03-14

**Authors:** Jose Carlos Noguera

**Affiliations:** Departamento de Ecología y Biología Animal, Universidad de Vigo, Vigo, Galicia, Spain

**Keywords:** Antioxidants, Oxidative stress, Predictive adaptive responses, Trade-offs

## Abstract

The hypothesis that oxidative damage accumulation can mediate the trade-off between reproduction and lifespan has recently been questioned. However, in captive conditions, studies reporting no evidence in support of this hypothesis have usually provided easy access to food which may have mitigated the cost of reproduction. Here, I test the hypothesis that greater investment in reproduction should lead to oxidative damage accumulation and telomere loss in domestic zebra finches *Taeniopygia guttata*. Moreover, since the change or fluctuation in diet composition between early and late postnatal period can impair the ability to produce antioxidant defences in zebra finches, I also tested if early nutritional conditions (constant vs fluctuating early diet) influenced the magnitude of any subsequent costs of reproduction (e.g., oxidative damage and/or telomere shortening). In comparison to pairs with reduced broods, the birds that had to feed enlarged broods showed a higher level of oxidative DNA damage (8-OHdG), but brood size had no effect on telomeres. Fluctuating early diet composition reduced the capacity to maintain the activity of endogenous antioxidants (GPx), particularly when reproductive costs were increased (enlarged brood). The decline in GPx in birds feeding enlarged broods was accompanied by a change in bill colouration. This suggests that birds with lower endogenous antioxidant defences might have strategically increased the mobilization of antioxidants previously stored in other tissues (i.e., bill and liver) and thus, preventing an excessive accumulation of damage during reproduction.

## Introduction

According to evolutionary theory, the immortal ‘Darwinian demon’ with an unlimited capacity to reproduce should exist only in the absence of constraints. This is because trade-offs between reproduction and other life history components such as longevity prevent living organisms from becoming such creatures (Harshman & Zera, 2007; Speakman, 2008). However, though such trade-offs are presumed to have a physiological basis (Zera & Harshman, 2001), our knowledge of the underlying mechanisms is still weak.

One physiological mechanism that has been hypothesised to underpin the negative effects of reproduction on lifespan is oxidative stress (reviewed in Metcalfe & Monaghan, 2013; Stier et al., 2012). Oxidative stress occurs when the level of oxidant molecules (generally referred as reactive oxygen species; ROS) exceeds the quenching capacity of the body’s antioxidant defence system and repair mechanisms, leading to oxidative damage (Halliwell & Gutteridge, 2015). This damage contributes to cellular ageing and the gradual deterioration of bodily function over time, as well as the whole organism senescence (Finkel & Holbrook, 2000; Murphy et al., 2011). Moreover, the accumulation of oxidative damage may also trigger other important cellular senescence pathways such as telomere shortening (Von Zglinicki, 2002). Telomeres are repeated tandem sequences of nucleotides that cap and protect linear chromosomes (Campisi et al., 2001). They shorten due to both the incomplete replication of the lagging DNA strand during cell division and because of oxidative damage to the telomeric DNA (Von Zglinicki, 2002). When a critically short telomere length is reached, cell senescence occurs (reviewed by Campisi et al., 2001; Xin, Liu & Songyang, 2008). Accumulating evidence indicates that individuals having shorter telomeres or a higher loss rate often have an increased risk of disease (Calado & Young, 2009) and shorter lifespan (Heidinger et al., 2012). As reproduction is associated with increased demands for various resources (i.e., energy and antioxidant compounds; Costantini, 2014; Speakman, 2008), it has been hypothesised that an experimentally increased reproductive effort will lead to more oxidative damage accumulation and potentially, telomere loss, and that both processes will contribute reducing lifespan (Metcalfe & Alonso-Alvarez, 2010; Monaghan, 2010; Speakman et al., 2015).

Intriguingly, although the hypothesised oxidative cost of reproduction has attracted the attention of many researchers in the last decade, experimental evidence still remains inconclusive (reviewed in Metcalfe & Monaghan, 2013). Indeed, it has been shown that reproduction can result in an increase (Alonso-Álvarez et al., 2010; Olsson et al., 2012; Stier et al., 2012), no change (Beaulieu et al., 2011; Markó et al., 2011; Nussey et al., 2009; Sudyka et al., 2016) or even a reduction (Blount et al., 2016; Costantini, Casasole & Eens, 2014; Garratt et al., 2011) in levels of oxidative damage. Similarly, evidence that reproduction can lead to significant telomere shortening is inconclusive, partly because of the paucity of studies (see i.e., Reichert et al., 2014; Sudyka et al., 2014). Recently, it has been suggested that the lack of consensus is caused by weaknesses in the experimental designs used to test the hypothesis (Metcalfe & Monaghan, 2013; Speakman et al., 2015). For instance, the majority of previous studies have based their conclusions on correlational data or from comparisons of reproducing vs non-reproducing individuals instead of effectively manipulating their reproductive investment (reviewed by Metcalfe & Monaghan, 2013). Moreover, many studies have been carried out in conditions where resources were freely accessible with minimal effort, which could have greatly mitigated any potential costs of reproduction. For instance, if food is easily accessible, then individuals facing a high reproductive investment could increase their consumption level, thereby avoiding the allocation trade-off between reproduction and self-maintenance (reviewed by Metcalfe & Monaghan, 2013).

If oxidative stress contributes to the cost of reproduction, the capacity to endogenously produce antioxidant defences (i.e., enzymatic antioxidants) as well as the ability to acquire, store and mobilise exogenous antioxidants (i.e., vitamins and carotenoid pigments) are likely to influence the magnitude of the oxidative cost of reproduction (Costantini, 2014; Metcalfe & Alonso-Alvarez, 2010). In this regard, the composition of the diet in early life in terms of antioxidant content may play an important role, since it can influence the way in which the antioxidant defence system operates later in life (Blount et al., 2003a; Noguera, Monaghan & Metcalfe, 2015; Romero-Haro & Alonso-Alvarez, 2015; Romero-Haro, Sorci & Alonso-Alvarez, 2016). For example, it has recently been shown that when diet composition in terms of antioxidant vitamins and mineral content is slightly lower than normal, young zebra finch (*Taeniopygia guttata*) nestlings can adjust the development of their antioxidant defence system (i.e., by increasing the production of some endogenous antioxidants and/or the absorption of exogenous vitamins) and compensate for such deficiencies (Noguera, Monaghan & Metcalfe, 2015). However, although potentially beneficial in the short term, theory predicts that these early phenotypic adjustments may be detrimental if (nutritional) conditions change later during development (reviewed by Nettle & Bateson, 2015). Indeed, when diet composition in terms of vitamin and mineral content fluctuates markedly between the early and late postnatal period, zebra finch nestlings suffer impairment in some components of their antioxidant system at adulthood (Noguera, Monaghan & Metcalfe, 2015). Having a reduced antioxidant capacity during adulthood may affect the development of different phenotypic traits (i.e., sexual traits; Romero-Haro & Alonso-Alvarez, 2015) but more importantly, it might also influence the magnitude of the cost of reproduction later in life as recently suggested (Romero-Haro, Sorci & Alonso-Alvarez, 2016).

In this study, I use zebra finches to test the hypothesis that oxidative damage accumulation and telomere loss are proximal costs of reproduction. I also test whether nutritional conditions experienced during their early life influence the magnitude of these costs. An experimental approach is used in which diet composition is either constant or fluctuating between early and late postnatal period, and reproductive effort is manipulated by alterations of clutch and brood size. To mimic the foraging costs in the wild (Metcalfe & Monaghan, 2013), I use a recently developed technique that forces the birds to physically exercise/fly to get their food. If reproduction carries oxidative costs, I predict a decrease in antioxidant defences (i.e., total antioxidant capacity and GPx level), an accumulation of oxidative DNA damage (i.e., 8-hydroxy-2-deoxyGuanosine) and greater loss of telomere length in parents rearing enlarged rather than reduced broods. Moreover, if growing on a fluctuating rather than a constant early diet composition induces long-term impairment in some components of the antioxidant defence system (Noguera, Monaghan & Metcalfe, 2015), the magnitude of the oxidative cost of reproduction should be higher in birds that were reared on a fluctuating rather than a constant diet.

## Material & Methods

### Animal housing and early life dietary manipulations

The study was conducted in the animal facilities at the University of Glasgow. I used adult male and female domestic zebra finches that were reared on different diet composition during their full postnatal period (from hatching to 90 days of age). A detailed description of the dietary treatments and housing conditions of the birds is described in Noguera, Monaghan & Metcalfe (2015). The main goal of that previous study was to assess the effect of diet during the postnatal growth period (from hatching to 90 days of age) on the development of the birds’ antioxidant defence system (see Noguera, Monaghan & Metcalfe, 2015). Briefly, from one day after hatching onwards, 57 zebra finch chicks (27 males and 30 females from 16 different families) were fed with a special seed mix composed by Proso and Finger millet in a ratio 1:1 (‘Control’ diet). At 40 days of age, half of the birds were kept on the same diet as during the first 40 days of life, and half were switched to a different diet (‘Vitamin-supplemented’ diet) that consisted in the same seed mix that birds received previously (Control) but now supplemented with a commercial vitamin mix (Magic Antistress Mix/Performax, Feed-Food Ltd, UK; a full description of the antioxidant content of the experimental diets and the commercial antioxidant supplement is provided in Noguera, Monaghan & Metcalfe, 2015). At 90 days, when birds were sexually mature, the dietary treatments ceased and all birds were fed with a standard aviary diet of mixed seeds (common millet, yellow millet and canary seed in an approximate ratio of 3:1:1; Johnson & Jeff, UK Johnson & Jeff, UK) and maintained in single-sex groups. This experimental design led to two experimental groups of birds; one where diet composition in terms of antioxidant and mineral content remained ‘constant’ (hereafter referred to as ‘constant early diet’ or ‘C’) during the full postnatal period and another where diet composition ‘fluctuated’ (F) between early and late postnatal period (hereafter referred to as ‘fluctuating early diet’ or ‘F’; a detailed chronogram of the experiment is shown in Supplemental Information 1). Importantly, such dietary manipulations influenced multiple aspects of the antioxidant defence system of the birds at early adulthood (90 days of age); F-birds exhibited lower levels of total antioxidant capacity in the blood and showed an impaired capacity to produce endogenous enzymatic antioxidants (GPx) compared to C-birds (see Noguera, Monaghan & Metcalfe, 2015 for further details). In this study, these two experimental groups of birds were used to investigate the influence of early dietary conditions (constant vs fluctuating early diet quality) on the magnitude of any potential oxidative costs linked to reproduction later in life (middle-age), when the birds had to breed for first time (see below).

### Housing and breeding effort manipulation

When the birds were approximately 2 years old (range 1.8–1.9 years), the males and females that were still alive were paired according to their early nutritional treatment in order to create experimental breeding pairs (*n* = 20 pairs, 10 C and 10 F) in which both members shared the same postnatal nutritional treatment (C or F). No bird had reproduced before the study. Pairs were then housed in cages (60 × 50 × 50 cm) equipped with an external nest box, coconut fibre as nesting material, and a cuttlefish bone (Johnson & Jeff, UK).

Although some previous studies have found some evidence in favour of oxidative costs of reproduction even when birds were fed *ad libitum* (Alonso-Alvarez et al., 2004; Wiersma et al., 2004), in my study I opted to make resources not superabundant as recently recommended (Metcalfe & Monaghan, 2013) by equipping each breeding cage with a high workload feeder similar to that described by Koetsier & Verhulst (2011). The feeder consisted of a transparent plastic container (20 × 15 × 5 cm) placed 30 cm above the ground and with two holes (ID 2.7 cm) in its front side from which the food (mixed seeds; Johnson & Jeff, UK) could be obtained by a bird when in energetically-demanding hovering flight (see Koetsier & Verhulst, 2011). Thus, in each feeding attempt, the birds were forced to fly 0.6 m and keep hovering in front of the feeder an average of 0.88 s (estimated from a group of 10 non-experimental birds that were filmed before the study). The aim of using high workload feeder was not to experimentally test the influence of foraging effort on reproductive costs but to ensure that food resources were not freely accessible with minimal effort (see. i.e., Metcalfe & Monaghan, 2013). Once a week, the birds also received Calcivet calcium supplement in the drinking water (Vetafarm, Wagga, NSW, Australia), a protein supplement (JE Haith, Cleethorpes, UK) and fresh vegetables. All birds were maintained at a temperature of 22 ± 1 °C and a photoperiod of 12 L: 12 D, as recommended in Olson et al. (2014). Nonetheless, note that this photoperiod was slightly different from that used during the first 100 days after hatching (see Noguera, Monaghan & Metcalfe, 2015). Nest boxes were checked daily from pairing, and the laying date of every new laid egg was recorded. A clutch was considered completed if no new eggs were laid for 4 days. One pair (C) did not reproduce and was therefore excluded from the analyses.

Since both egg incubation and chick rearing are energetically demanding phases of reproduction in birds (Monaghan & Nager, 1997), either incubating a larger clutch or rearing a larger brood has the potential to increase levels of oxidative stress in the parents. Hence, in contrast to previous studies investigating hypothesised oxidative costs of reproduction (i.e., Alonso-Alvarez et al., 2004; Costantini, Casasole & Eens, 2014; Reichert et al., 2014), in this experiment I decided to manipulate both clutch and brood size (hereafter referred to as simply ‘brood size’). To that end, four days after the last egg in a clutch was laid, clutch size was reduced in half of the experimental breeding pairs (either C or F) and enlarged in the other half by swapping the whole clutch with 2 eggs (‘reduced’ group) or 5 eggs (‘enlarged’ group) respectively, all of these extra eggs coming from a set of non-experimental pairs (*n* = 40). This procedure allowed me to disrupt the natural covariation between parental quality and clutch/brood size. The original clutch size was not significantly different between experimental groups (Generalized Lineal Model (GLM): nutritional treatment × breeding effort: *F*_1,15_ = 1.773, *p* = 0.203; breeding effort: *F*_1,16_ = 0.001, *p* = 0.981; nutritional treatment: *F*_1,17_ = 0.700, *p* = 0.414). Nests of incubating birds were inspected daily, starting a few days before the expected hatching date. To maintain constant clutch size and brood size, one day after the last chick in a nest hatched, any unhatched egg was inspected. If it was unfertilised or the embryo was dead, then it was replaced by an extra chick from a non-experimental pair that hatched within ±24 h. Chick mortality did not differ between experimental groups during the experiment (Generalized Linear Mixed Model (GLMM) controlling for nest of origin: breeding effort: breeding effort × nutritional treatment: *F*_1,64_ = 1.483, *p* = 0.228; *F*_1,65_ = 0.071, *p* = 0.790; nutritional treatment: *F*_1,66_ = 0.300, *p* = 0.586;). This experimental design resulted in four different treatment groups that differed in the early diet of the adult birds (C or F) and the breeding effort (incubation and chick rearing) they had to cope with during adulthood (enlarged or reduced) (see Fig. S1 for further details).

All the adult breeders were weighed (±0.01 g), and their blood sampled just before (pairing day) and after (when chicks aged 24 days) reproduction. Blood samples, approx. 90 µl, were taken from the brachial vein with heparinised capillary tubes. The time elapsed between the two blood samples did not differ among experimental groups (Linear mixed effect model-LMM; nutritional treatment × brood size: *F*_1,15_ = 1.674, *p* = 0.255; nutritional treatment: *F*_1,16_ = 0.100, *p* = 0.756; brood size: *F*_1,17_ = 0.763, *p* = 0.394). Blood samples were maintained on ice and then centrifuged (2,000 g, 10 min at 4°) to separate plasma from red blood cells (RBCs). Several aliquots were made from both plasma and RBCs samples and then stored at −80C. In addition to body mass, bill colour was also measured in all birds before and after the breeding experiment on a scale of 1 (light orange) to 9 (dark red) using the standard colour chips previously described in Blount et al. (2003a) and Blount et al. (2003b).

### Testing the effect of brood size manipulation on parental foraging effort

To assess the effectiveness of brood size in increasing foraging costs, parental foraging effort was assessed when the broods were 18 days of age, which approximately corresponds to the middle point of the rearing period (Zann & Bamford, 1996). For logistic reasons, parental foraging effort was only assessed in a subsample of the experimental pairs (5 reduced and 5 enlarged; *n* = 20 birds). The breeding pairs were observed for 4 h (between 10.00 and 14.00 h) from behind a screen placed approx. 1.5 m away from the cage and the total number of feeding flights performed by both adults (male and female) was recorded. Parental foraging effort was then expressed as the number of feeding flights each bird performed per hr.

### Measurements of oxidative stress and telomere length

#### Non-enzymatic antioxidant defences

Total non-enzymatic antioxidant capacity (TAC) was measured in plasma samples using the method described by Erel (2004). The main antioxidants contributing to this assay are hydrophilic antioxidants such as the—SH group of proteins, ascorbate and uric acid and, to a lesser extent, vitamin-E and other hydrophobic antioxidants. The assays were calibrated with Trolox (*R*^2^ > 0.98 in all cases) and the level of TAC expressed as millimoles of Trolox equivalent per litre. All samples were assayed in duplicated (average intra and inter-assay CV of 5.4% and 7.3%, respectively).

#### Enzymatic antioxidant defences

Enzymatic antioxidant defences were assessed by analysing the red blood cell (RBC) activity of glutathione peroxidase (GPx), the most important and widespread intracellular antioxidant enzyme (Arthur, 2001). GPx catalyses the reduction of hydroxyperoxides and hydrogen peroxide. GPx activity was measured using a commercial kit (Cayman Chemical, Ann Arbor, MI, US; Catalog Number 703102), and following the manufacturer’s instructions. All samples were run in duplicate (average intra and inter-assay CV of 7.1% and 5.7%, respectively) and GPx activity was expressed as nmol/min/mg protein.

#### Oxidative DNA damage

Oxidative DNA damage was assessed by measuring the amount of 8-hydroxy-2-deoxyGuanosine (8-OHdG) in RBCs DNA, using commercial kits (EpiQuik TM 8-OHdg DNA damage Quantification Direct Kit; Epigentek Group Inc. Farmingdale, USA; Catalog Number P-6003). 8-OHdG is widely used as a marker of oxidative DNA damage, has important mutagenic potential, and has been related to the ageing process and cancer (Best, 2009). For the analyses, DNA was first extracted from RBCs with commercial kits (Macherey-Nagel, Bethlehem, PA, USA). Samples were assayed in duplicate (average intra and inter-assay CV of 8.0 and 8.7%, respectively) and calibrated with the 8-OHdG standard (all *R*^2^ > 0.99). Oxidative DNA damage levels were expressed as ng of 8-OHdG.

#### Telomere length analyses

Telomere length was quantified by the qPCR method described in (Criscuolo et al., 2009). The relative telomere length of each sample was measured by determining the ratio (T/S) of telomere repeat copy number (T) to single control gene copy number (S), relative to a reference sample. Glyceraldehyde-3-phosphate dehydrogenase (GAPDH) was used as the single control gene. A detailed description of the PCR conditions and validation of the protocol in zebra finches is provided in (Criscuolo et al., 2009). In all plates, reaction efficiencies (%) were within an acceptable range (109, 94.7, 96.6 and 92.7, 95.5, 93.9 for telomere and GAPH, respectively). All samples were run in triplicate and the average T/S ratios were calculated controlling for plate efficiency (Pfaffl, 2001). The average intra- and inter-plate coefficient of variation of the T/S values were 0.04% and 0.05%, respectively.

### Statistical analysis

All statistical analyses were carried out using IBM SPSS 22 for Windows. I first used linear mixed effect models (LMM) to test the effectiveness of the brood manipulation in increasing parental foraging effort (feeding flights of each bird/hr; log-transformed). In the model, brood size (reduced or enlarged), sex of the adult and their interaction were included as fixed factors while the identity of the cage was included as random factor. Secondly, I used linear models (LM) to analyse pre-breeding (pairing day) between-treatment differences in body mass, bill colour, antioxidant defences (TAC level and GPx activity), oxidative DNA damage level and telomere length. The models included the nutritional treatment (C or F), brood size manipulation (reduced or enlarged), and sex as fixed factors as well as all their possible two- and three-way interactions. I then analysed the effect of dietary treatments and breeding effort on the change (final minus initial values) in traits over the course of the breeding season. These models included the same fixed factors as the pre-breeding models but also initially included the identity of the cage as a random factor. However, the random factor had no significant effect (all *p* > 0.35) and hence, was removed in subsequent analyses. Since pre-breeding values of bill colouration differed between nutritional treatment groups (see ‘Results’), prior to the analyses the change in bill colouration values were corrected for the regression to the mean as described in Verhulst et al. (2013). In addition, I also calculated the Pearson’s (r) correlation coefficient between the change in GPx activity and bill colouration, and parental feeding rate and the change in oxidative DNA damage. These secondary analyses allowed me to assess (1) whether birds with reduced ability to produce endogenous antioxidants mobilised previously stored exogenous antioxidants in the body (i.e., bill) and (2) whether a higher accumulation of oxidative damage might be the results of a higher parental feeding effort. All the above models were simplified by removing non-significant terms (in backward deletion procedure), starting from three-way interactions. Similar results were obtained when models were not simplified (see Tables S1 and S2 for a detailed description of full models). The Satterthwaite approximation was used for the estimation of degrees of freedom, and *post hoc* comparisons were carried out using Tukey’s *post hoc* test. The residuals of all models were tested for normality (Kolmogorov–Smirnov test). Data are presented as means ± se, significant level was set at *P* = 0.05. Mean ± se for each experimental group and oxidative stress marker is also provided in Table S3).

**Ethical Note:** All procedures carried out in this study were under the jurisdiction of a UK Home Office project license (60/4109) governed by the UK Animals Scientific Procedures Act 1986. All applicable institutional and/or national guidelines for the care and use of animals were followed.

## Results

### Effectiveness of the brood manipulation in increasing parental foraging effort

**Figure 1:**
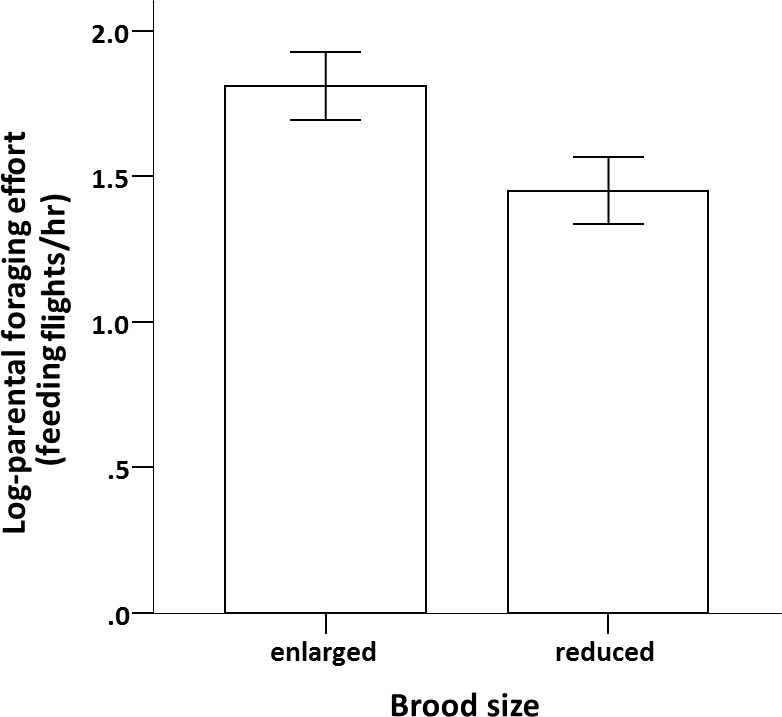
Effect of brood size on parental reproductive effort. Effect of brood size manipulation on individual parental foraging effort (log-feeding flights/hr; mean ± se) when broods were 18 days of age.

The adult birds that had enlarged broods performed, on average, more than double the number of feeding flights compared to those with reduced broods (*F*_1,18_ = 19.229, *p* < 0.001; Fig. 1). Foraging effort was similar between sexes in both experimental groups (sex × brood size: *F*_1,16_ = 3.124, *p* = 0.096; sex: *F*_1,17_ = 0.009, *p* = 0.927).

### Effects of early life nutrition and brood size manipulation on adult body mass and bill colouration

At the beginning of the experiment (day of pairing), body mass was similar among experimental groups and sexes (see Table S4 for further details). However, bill colour differed between nutritional treatments; birds of both sexes reared on a fluctuating (F) diet composition had redder bills at the beginning of the experiment (*F*_1,35_ = 6.650, *p* = 0.014). Also, bill colour differed between sexes, with males having redder bills than females (*F*_1,35_ = 59.981, *p* < 0.001). None of the other considered variables had a significant effect on bill colour (see Table S4).

**Figure 2:**
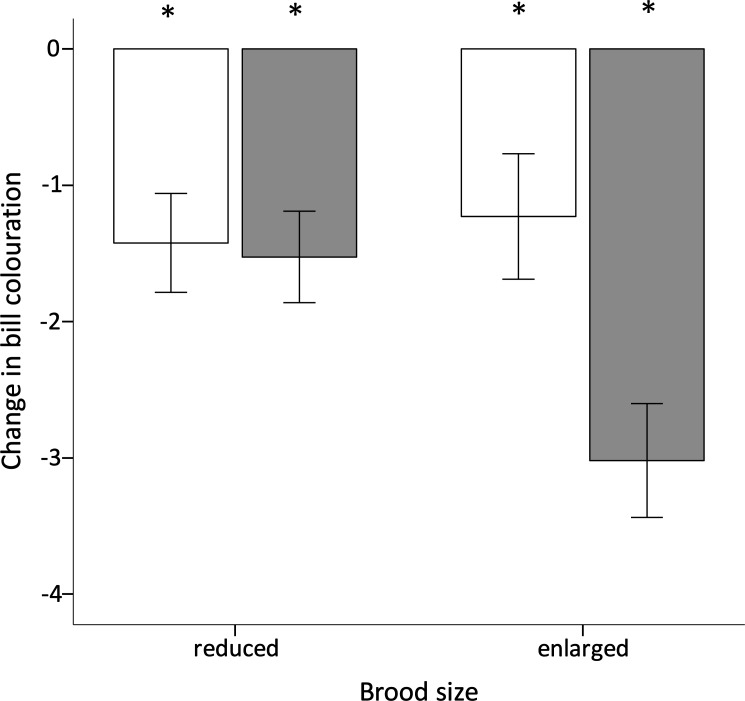
Effect of dietary treatment and reproductive effort on bill colouration. Change in bill colouration during reproduction (mean ± se) of zebra finches reared on a constant (white bars) or fluctuating (grey bars) early availability of dietary antioxidants in relation to breeding effort. Negative values indicate a loss of redness; changes significantly different from zero (one sample *t*-test; *p* < 0.05) are denoted with an asterisk ‘*’.

**Figure 3:**
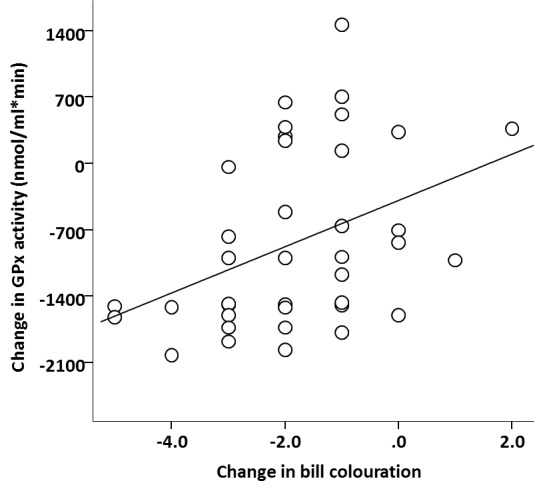
Relationship between variation in bill colouration and GPx activity. Change in bill colouration in relation to change in GPx activity during reproduction. The line represent an adjusted regression line.

During reproduction, all birds lost body mass (intercept = − 1.394 g, *F*_1,37_ = 36.370, *p* < 0.001), but the loss was similar in magnitude among experimental groups and sexes (Table 1). However, early nutritional treatment and brood size manipulation had a significant interactive effect on the change in bill colouration (Table 1). Among birds with a reduced brood, bill colouration decreased over the breeding season in both C- and F-birds but the magnitude was similar between the two groups (Tukey’s *post hoc* test: *p* = 0.938; Fig. 2). However, when the birds were forced to raise an enlarged brood, F-birds lost significantly more bill colour than C-birds (Tukey’s *post hoc* test: *p* = 0.005). This reduction in bill colour was significantly greater than in any other experimental group (*p* > 0.125 for all other Tukey’s *post hoc* tests). The change in bill coloration also differed between sexes; females lost significantly more bill colouration than males (Table 1). The analyses also revealed that the change in bill colouration was positively correlated with the change GPx activity (Pearson correlation coefficient: *r* = 0.39, *p* = 0.015; Fig. 3).

**Table 1:** Factors influencing body mass and bill colouration. Results of linear mixed models on the change (Δ) in adult body mass and bill colouration during reproduction. Variables retained in the final model are shown in bold. For removed terms, significance levels are those when terms were dropped from the model.

Source of variation	Δ Body mass				Δ Bill colour			
	Estimate	*F*	*d*.*f*._*n*,*d*_	*p*	Estimate	*F*	*d*.*f*._*n*,*d*_	*p*
Intercept	**−1.182**				**0.989**			
Nutritional treatment (C)	0.327	0.491	1,35	0.488	**0.103**	**6.643**	**1,33**	**0.015**
Brood size (enlarged)	0.154	0.106	1,34	0.747	**−1.492**	**3.124**	**1,33**	**0.086**
Sex (female)	−0.425	0.841	1,36	0.365	**−1.074**	**8.641**	**1,33**	**0.006**
Nutritional treatment × brood size	−1.120	1.407	1,33	0.244	**1.686**	**5.270**	**1,33**	**0.028**
Nutritional treatment × sex	0.743	0.616	1,32	0.438	0.525	0.507	1,32	0.482
Brood size × sex	0.427	0.198	1,31	0.660	−0.282	0.142	1,31	0.709
Nutritional treatment × brood size × sex	−1.261	0.421	1,30	0.521	0.303	0.040	1,30	0.844

### Effect of early life nutrition and brood size manipulation on markers of oxidative stress and telomere length

**Figure 4:**
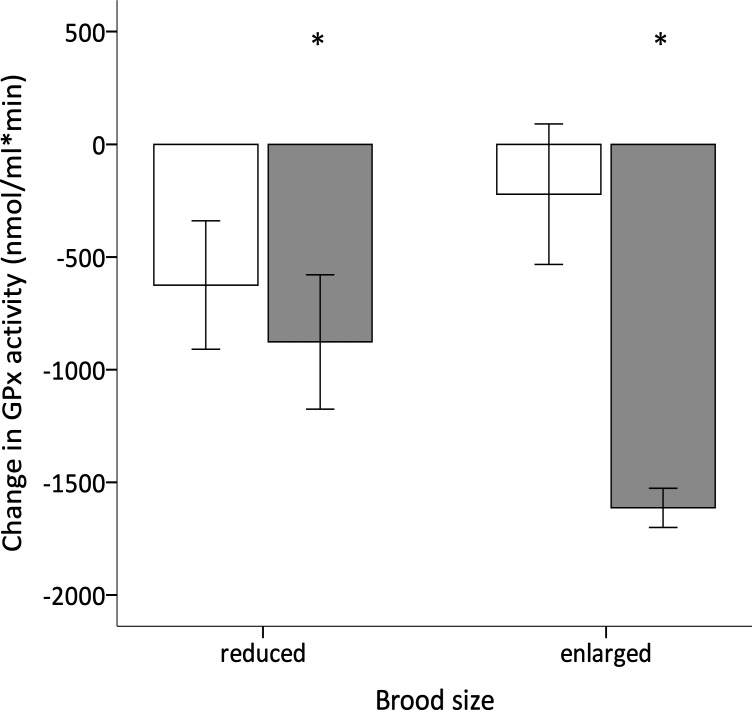
Effect of dietary treatment and reproductive effort on antioxidant defences. Change in GPx activity during reproduction (mean ± se) of zebra finches in relation to breeding effort (enlarged or reduced clutch and brood) and whether they were reared on a constant (white bars) or fluctuating (grey bars) early availability of dietary antioxidants. Changes significantly different from zero (one sample *t*-test; *p* < 0.05) are denoted with an asterisk ‘*’.

**Figure 5:**
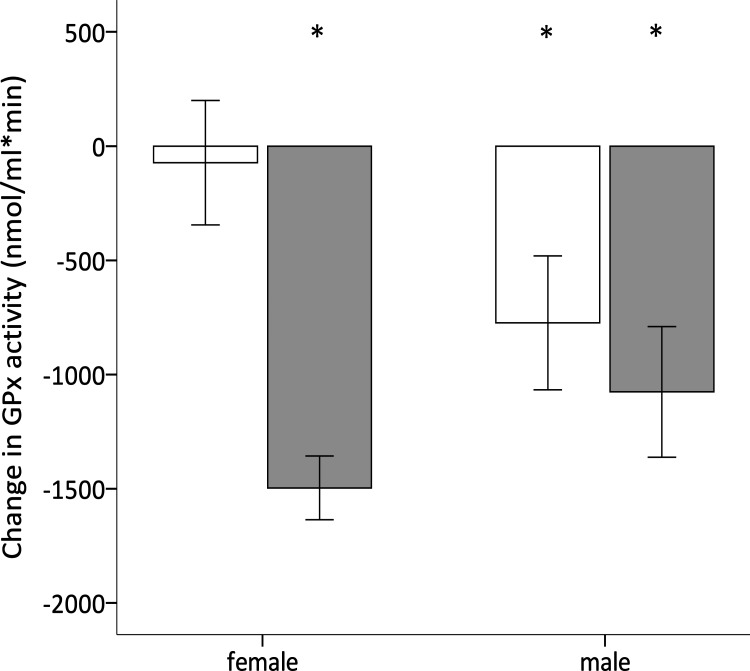
Effect of dietary treatment and sex on antioxidant defences. Change in GPx activity during reproduction (mean ± se) of adult zebra finches reared on a constant (white) or fluctuating (grey) early availability of dietary antioxidants. Changes significantly different from zero (one sample *t*-test; *p* < 0.05) are denoted with an asterisk ‘*’.

GPx activity, TAC and oxidative DNA damage levels, and telomere lengths were similar among experimental groups and sexes prior to reproduction (see Table S4 for further details). Over the reproductive period there was no effect of the early nutritional treatment, brood size manipulation, sex or their interactions on the change in TAC (Table 2), which did not significantly differ from zero (intercept =0.148, *F*_1,37_ = 3.354, *p* = 0.075). However, the change in GPx over the same period was affected by the brood size manipulation, although the effect depended on the early nutritional treatment of the birds (Table 2; Fig. 4). Specifically, whereas C- and F-birds showed a similar reduction in their GPx activity when raising a reduced brood (Tukey’s *post hoc* test: *p* = 0.895), in the enlarged brood group F-birds experienced a significantly greater decline in GPx activity compared to C-birds (Tukey’s *post hoc* test: *p* = 0.001; Fig. 4). In addition, the change in GPx activity was affected by the sex of the birds although the effect also differed according to the early nutritional treatment (Table 2); whereas in males the change in GPx did not depend on whether they had experienced a fluctuating or constant early diet (Tukey’s *post hoc* tests: *p* = 0.877), females showed a significant decline in GPx if they were reared on a fluctuating compared to a constant early diet (Tukey’s *post hoc* test: *p* = 0.002; Fig. 5).

**Table 2:** Factors influencing oxidative stress markers. Results of linear mixed models (LMM) on the change (Δ) in antioxidant defences (TAC and GPx), oxidative damage and telomere length over the period of reproduction. Variables retained in the final model are shown in bold. For removed terms, significance levels are those when terms were dropped from the model.

	ΔTAC				ΔGPx				ΔDNA damage				ΔTelomere length			
*Source of variation*	Estimate	*F*	*d*.*f*._*n*,*d*_	*p*	Estimate	*F*	*d*.*f*._*n*,*d*_	*p*	Estimate	*F*	*d*.*f*._*n*,*d*_	*p*	Estimate	*F*	*d*.*f*._*n*,*d*_	*p*
Intercept	**0.056**				**−667.055**				**0.233**				**−0.034**		
Nutritional treatment (C)	0.174	1.170	1,36	0.287	**−308.429**	**11.038**	**1,32**	**0.002**	−0.301	0.103	1,34	0.750	0.089	1.375	1,36	0.249
Brood size (enlarged)	0.121	0.550	1,34	0.464	**−736.223**	**0.453**	**1,32**	**0.506**	**3.110**	**11.556**	**1,36**	**0.002**	−0.064	0.694	1,35	0.411
Sex (female)	0.122	0.569	1,35	0.456	**−420.331**	**0.325**	**1,32**	**0.573**	−0.381	0.170	1,35	0.682	0.021	0.074	1,34	0.788
Nutritional treatment × brood size	0.533	0.326	1,32	0.572	**1139.293**	**5.300**	**1,32**	**0.028**	2.500	1.808	1,33	0.188	−0.147	0.887	1,33	0.353
Nutritional treatment × sex	−0.184	2.800	1,33	0.104	**1121.679**	**5.171**	**1,32**	**0.030**	−1.023	0.298	1,32	0.589	0.019	0.015	1,31	0.903
Brood size × sex	0.066	0.041	1,31	0.840	236.923	0.224	1,31	0.639	−0.825	0.189	1,31	0.667	−0.030	0.036	1,32	0.850
Nutritional treatment × brood size × sex	0.773	1.408	1,30	0.245	−1003.76	1.004	1,30	0.324	−4.267	1.266	1,30	0.269	0.205	0.396	1,30	0.534

**Figure 6:**
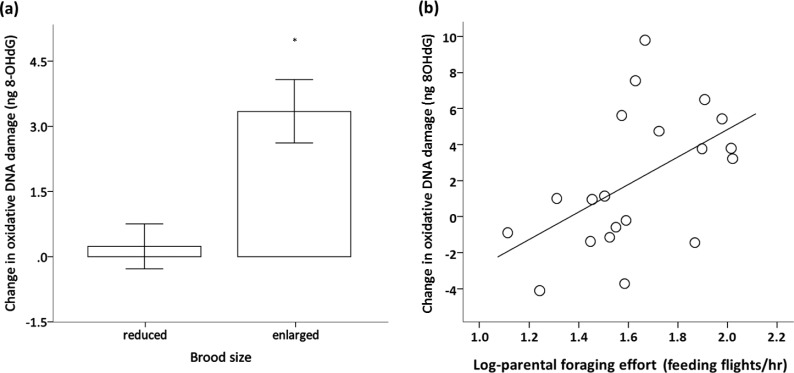
Effect of brood size and foraging effort on oxidative DNA damage. Change in oxidative DNA damage (ng of 8-OHdG; mean ± se) of adult zebra finches in relation to (A) brood size manipulation and (B) their individual foraging effort. In (A) changes significantly different from zero (one sample *t*-test; *p* < 0.05) are denoted with an asterisk ‘*’.

Importantly, the brood size manipulation also affected oxidative DNA damage levels (Table 2). Oxidative DNA damage significantly increased over the reproductive period in the birds that had to incubate and raise an enlarged brood in comparison to the birds with a reduced brood (Fig. 6A). This effect of brood size manipulation on DNA damage did not depend on early nutritional treatment and was similar between the sexes (Table 2). The nutritional treatment, brood size, sex and their interactions had no effect on the change in telomere length (all *p* > 0.249; see Table 2) which did not significantly differ from zero (intercept = − 0.034, *F*_1,37_ = 0.796, *p* = 0.378). Secondary analyses revealed that the accumulation of oxidative DNA damage was positively related to parental feeding effort (Pearson correlation coefficient: *r* = 0.51, *p* = 0.021; Fig. 6B).

## Discussion

The role of oxidative stress as a mediator underlying a cost of reproduction has recently been extensively debated (Blount et al., 2016; Metcalfe & Monaghan, 2013; Selman et al., 2012). In this study, I show that zebra finches that had to raise an experimentally enlarged brood had a higher oxidative DNA damage (8-OHdG) level in comparison to birds rearing a reduced brood. I also show that fluctuating diet composition early in life reduces the capacity to maintain the activity of endogenous antioxidants (GPx), particularly when reproduction is demanding (e.g., when rearing an enlarged brood). However, the decline in GPx activity in these birds was not mirrored in increased DNA damage accumulation, suggesting that the birds that had experienced a fluctuating early diet composition and had to face a highly demanding reproduction activated other antioxidant defence mechanisms.

Many of the studies examining the relationship between reproduction and oxidative stress have been conducted in captive conditions in which foraging costs were minimal (see i.e., Metcalfe & Monaghan, 2013 and references therein). In that situation, animals could potentially increase their food intake with minimum energetic expenditure and so may not have needed to reduce their investment in somatic maintenance (Metcalfe & Monaghan, 2013). Due to using high-workload feeders in my experiment, male and female zebra finches rearing enlarged broods had to perform more feeding flights than birds rearing a reduced brood. Short routine flights accompanied by numerous take-offs are energetically expensive (Koetsier & Verhulst, 2011; Nudds & Bryant, 2000) and could have led to increased levels of oxidative stress (Costantini, Mirzai & Metcalfe, 2012) presumably via different mechanisms such as by increasing ROS production, reducing the level of antioxidant defences or leading to haemoglobin auto-oxidation (reviewed by Cooper et al., 2002). Indeed, this is in agreement with the observed covariation between parental feeding effort and oxidative DNA damage, and the fact that only the enlarged brood size group of birds seemed to have accumulated more oxidative DNA damage (8-OHdG) over the course of reproduction. Thus, my results suggest that, at least in part, the difference in oxidative DNA damage accumulation between birds rearing reduced and enlarged broods might be attributed to differences in foraging effort. However, since foraging effort was not experimentally manipulated in my experiment, further studies should be carried out to firmly establish such possibility. In addition, although my results strongly suggest that rearing a large brood can be oxidatively costly, it is important to emphasise that my study lacked a non-manipulated (control) group. Hence, the direction of the effect observed in oxidative DNA damage could potentially have followed the opposite direction; birds rearing a reduced brood experiencing a significant reduction in oxidative DNA damage. Although such possibility is less likely since most studies employing brood size manipulation have demonstrated that non-manipulated controls usually do not bring about any significant effects in comparison to reduced broods (Santos & Nakagawa, 2012), future studies should take it into account when interpreting the results of this study.

The apparent higher accumulation of oxidative DNA damage of birds rearing enlarged broods would support previous studies in the same species (Alonso-Alvarez et al., 2004; Wiersma et al., 2004). However, in contrast to my study, Alonso-Alvarez et al. (2004) and Wiersma et al. (2004) did not manipulate clutch size, suggesting that it is the chick-feeding period and not the incubation period that is the breeding phase that is more likely to be oxidatively costly for an adult breeder. Intriguingly, a recent study carried out in the same species found no evidence supporting the oxidative costs of reproduction (Sudyka et al., 2016). Differences in experimental conditions between studies could provide some explanations for such contrasting results. For instance, although in Sudyka et al. (2016), birds rearing enlarged broods were also challenged by a low ambient temperature, it might be possible that neither the brood manipulation nor the lower ambient temperature used was enough to stimulate a significant accumulation of oxidative damage. Moreover, since oxidative stress markers are not equally sensitive to and respond in the same way to changes in oxidative status (Speakman et al., 2015), it is also plausible that in some previous studies even if rearing an enlarged brood was oxidative costly for the birds, the markers used were not enough sensitive to track the variation in parental oxidative status.

Two recent studies in songbirds have reported a reduction in telomere length as a consequence of a greater investment in reproduction (Reichert et al., 2014; Sudyka et al., 2014), but I did not find any effect of brood size manipulation on telomere dynamics. At first glance, this result is surprising when considering that (1) the level of oxidative DNA damage increased during reproduction in the enlarged group of birds and (2) the accumulation of oxidative damage seems to be involved in telomere loss, at least *in vitro* (Kawanishi & Oikawa, 2004; Von Zglinicki, 2002). Nonetheless, it is plausible that even if a high investment in reproduction resulted in a significant accumulation of oxidative DNA damage, the magnitude of this damage was too small or too short-lived to induce an observable reduction in telomere length. Indeed, this might explain why zebra finches rearing enlarged broods suffer a significant decline in telomere length when telomere loss is assessed for a longer time period during the chick rearing period (Reichert et al., 2014). However, in this previous study the authors did not find a significant accumulation of oxidative DNA damage during the same time period. Hence, results in Reichert et al. (2014) partially contrasts with my results and suggest that the relationship between oxidative stress and telomere loss is more complex than suggested by *in vitro* studies, even within the same species. Nonetheless, differences between studies should be analysed with caution since housing conditions of the birds were not exactly the same between studies (i.e., temperature, light:dark cycle, etc.) which may have affected the results (reviewed by Griffith et al., 2017).

GPx level declined substantially when the birds were exposed to a fluctuating diet early in life and then had a high reproductive investment (i.e., enlarged brood). This supports the idea that oxidative cost or reproduction may depend on early developmental conditions, especially those able to influence oxidative status (Romero-Haro, Sorci & Alonso-Alvarez, 2016). However, early dietary conditions had no influence on GPx among adult birds with reduced broods. Hence, the effects of a fluctuating early diet composition on GPx seem to be context-dependent, becoming evident only under certain situations where the cell redox balance might be profoundly altered (Arthur, 2001). A change in antioxidant allocation strategies as a consequence of the early life diet might explain these results. For instance, although birds in the fluctuating diet group had a redder bill colouration suggesting that they could have enough antioxidants for the development of secondary sexual characters, they might alternatively have prioritised the use of dietary antioxidants in the development of secondary sexual characters instead of cell antioxidant protection. Although this allocation strategy could be advantageous in the short-term i.e., by increasing sexual attractiveness and mating success; (Zann & Bamford, 1996), it is likely to have reduced the capacity of the F-birds to efficiently detoxify any excess of ROS (Surai, 2002), especially when rearing an enlarged brood. Higher levels of ROS, however, may have reduced GPx synthesis and activity by blocking key RNA binding proteins (i.e., SBP2) and reducing the incorporation of selenocysteine into the active sites of the GPx enzyme (Cheng et al., 1999; Lei, Cheng & McClung, 2007; Papp et al., 2006), thus explaining the observed variation in GPx activity. Alternately, the variation in GPx could be the result of changes in other unmeasured intracellular antioxidants, particularly those involved in the regeneration and maintenance of GPx activity (i.e., glutathione; Arthur, 2001; Lei, Cheng & McClung, 2007).

The effect of a fluctuating early diet on GPx was especially marked in females. This result is interesting given that foraging effort was similar among the sexes during the chick rearing period. However, the F-diet females were paired with males showing an initial redder bill colouration, a sexually selected trait in this species (Zann & Bamford, 1996). This may have resulted in a higher incubation effort of the females (Gorman, Arnold & Nager, 2005) and therefore, a greater energy expenditure (Monaghan & Nager, 1997) that may have imposed greater antioxidant demands. Indeed, sexual differences in energy expenditure and GPx activity during reproduction has previously been described in the zebra finch (Wiersma et al., 2004).

Assuming that a greater decline in GPx activity increased the likelihood of birds to accumulate more oxidative damage (Arthur, 2001; Brigelius-Flohé & Maiorino, 2013), then the question is why the variation in GPx activity did not match with the observed variation in oxidative DNA damage. It is likely that when facing a high reproductive effort, birds with reduced GPx activity have used diverse mechanisms to avoid a disproportionate accumulation of oxidative damage. One plausible mechanism might have been a major mobilisation of previously-stored antioxidants in other tissues and organs (i.e., skin, fat or liver; Costantini & Dell’Omo, 2006; Pérez, Lores & Velando, 2010; Surai, 2002). Indeed, this fits with the observed decline in bill colouration and the positive covariation between the change in GPx activity and bill colouration. The red-orange bill colour of zebra finches primarily results from high concentrations of metabolically derived carotenoids (Blount et al., 2003b), pigments that if mobilised may have contributed to increase the birds’ antioxidant protection (Martinez, 2009; Martínez, 2013; but see Costantini & Møller, 2008; Surai, 2002). Although some data suggest that bill carotenoids could be mobilised under situations of high oxidative stress (Rosenthal et al., 2012) and that they reflect the level of antioxidants stored in other body components (i.e., liver; McGraw & Toomey, 2010), empirical evidence to date is unclear and more experimental work is still needed to corroborate this hypothesis.

In conclusion, I have shown that captive birds facing a more costly reproductive event accumulated more oxidative DNA damage. The results also suggest that when diet composition in terms of antioxidant and mineral content markedly fluctuate during postnatal development, some components of the adult antioxidant defence system can be impaired during reproduction, but only when breeding effort is increased. When this situation occurs, the results further suggest that individuals might activate other defence mechanisms in order to slow down the rate of oxidative damage accumulation (i.e., mobilising previously stored antioxidants in other body components). Future studies should investigate whether early life dietary conditions may also influence the expression of other antioxidant-depend phenotypic traits such as those involved in sexual selection (i.e., signal expression and sperm quality).

##  Supplemental Information

10.7717/peerj.3094/supp-1 Supplemental Information 1 Supplemental Information (SI) Statistical details and protocols

## Additional Information and Declarations

### Data Availability

The following information was supplied regarding data availability: Data are deposited in the Figshare digital repository (URL): https://figshare.com/s/61dbf3b4c4b03da9607f.
