# Supplementary material for: Interacting effects of early dietary conditions and reproductive effort on the oxidative costs of reproduction
Source: PeerJ. 2017 Mar 14;5:e3094. doi: 10.7717/peerj.3094 (PMC5354074; doi:10.7717/peerj.3094)
Supplement: Supplemental Information 1 — Statistical details and protocols [file peerj-05-3094-s001.docx]

**INTERACTING EFFECTS OF EARLY DIETARY CONDITIONS AND REPRODUCTIVE EFFORT ON THE OXIDATIVE COSTS OF REPRODUCTION**

Noguera, Jose C.

SUPPLEMENTAL INFORMATION (SI)

**Figure S1**. Chronogram of the experiment


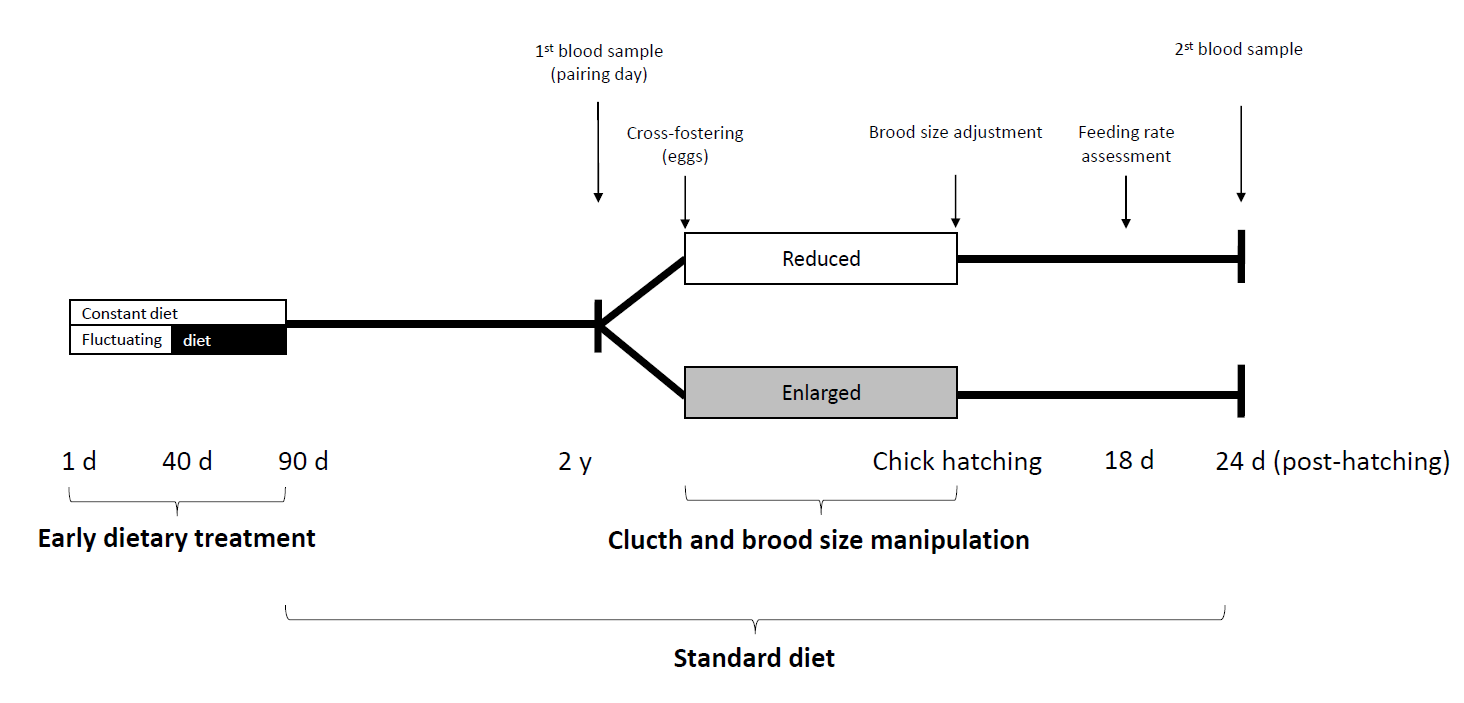


**Table S1**. Results of Full linear mixed models on the change (Δ) in adult body mass and bill colouration during reproduction.

|  | Δ Body mass | | | | Δ Bill colour | | | |
| --- | --- | --- | --- | --- | --- | --- | --- | --- |
| *Source of variation* | estimate | F | d.f._n,d_ | p | estimate | F | d.f._n,d_ | p |
| Intercept | -1.270 |  |  |  | -0.974 |  |  |  |
| Nutritional treatment (C) | 0.212 | 0.578 | 1,30 | 0.453 | -0.071 | 6.171 | 1,30 | 0.019 |
| Brood size (enlarged) | 0.200 | 0.149 | 1,30 | 0.703 | -1.271 | 2.902 | 1,30 | 0.099 |
| Sex (female) | -1.425 | 0.957 | 1,30 | 0.336 | -1.105 | 7.974 | 1,30 | 0.008 |
| Nutritional treatment x brood size | -0.490 | 1.330 | 1,30 | 0.258 | 1.535 | 4.895 | 1,30 | 0.035 |
| Nutritional treatment x sex | 1.435 | 0.686 | 1,30 | 0.414 | 0.349 | 0.431 | 1,30 | 0.516 |
| Brood size x sex | 1.095 | 0.229 | 1,30 | 0.636 | -0.443 | 0.146 | 1,30 | 0.705 |
| Nutritional treatment x brood size x sex | -1.261 | 0.421 | 1,30 | 0.521 | 0.303 | 0.040 | 1,30 | 0.844 |

**Table S2**. Results of Full linear mixed models (LMM) on the change (Δ) in antioxidant defences (TAC and GPx), oxidative damage and telomere length over the period of reproduction.

|  | ΔTAC | | | | ΔGPx | | | | ΔDNA damage | | | | ΔTelomere length | | | |
| --- | --- | --- | --- | --- | --- | --- | --- | --- | --- | --- | --- | --- | --- | --- | --- | --- |
| *Source of variation* | estimate | F | d.f._n,d_ | p | estimate | F | d.f._n,d_ | p | estimate | F | d.f._n,d_ | p | estimate | F | d.f._n,d_ | p |
| Intercept | -0.081 |  |  |  | -453.631 |  |  |  | 1.456 |  |  |  | -0.132 |  |  |  |
| Nutritional treatment (C) | 0.215 | 1.315 | 1,30 | 0.261 | -580.712 | 10.772 | 1,30 | 0.003 | -2.219 | 0.156 | 1,30 | 0.695 | 0.209 | 1.233 | 1,30 | 0.276 |
| Brood size (enlarged) | 0.390 | 0.606 | 1,30 | 0.442 | -1120.267 | 0.442 | 1,30 | 0.511 | 1.053 | 10.148 | 1,30 | 0.003 | 0.082 | 0.538 | 1,30 | 0.469 |
| Sex (female) | 0.032 | 0.524 | 1,30 | 0.475 | -847.179 | 0.226 | 1,30 | 0.638 | -0.639 | 0.173 | 1,30 | 0.680 | 0.086 | 0.090 | 1,30 | 0.767 |
| Nutritional treatment x brood size | -0.571 | 0.320 | 1,30 | 0.576 | 1641.174 | 5.172 | 1,30 | 0.030 | 4.634 | 1.739 | 1,30 | 0.197 | -0.250 | 0.818 | 1,30 | 0.373 |
| Nutritional treatment x sex | 0.127 | 2.490 | 1,30 | 0.125 | 1666.245 | 5.402 | 1,30 | 0.027 | 1.189 | 0.248 | 1,30 | 0.622 | -0.089 | 0.007 | 1,30 | 0.934 |
| Brood size x sex | -0.343 | 0.018 | 1,30 | 0.894 | 768.326 | 0.283 | 1,30 | 0.599 | 1.433 | 0.136 | 1,30 | 0.715 | -0.137 | 0.046 | 1,30 | 0.831 |
| Nutritional treatment x brood size x sex | 0.773 | 1.408 | 1,30 | 0.245 | -1003.761 | 1.004 | 1,30 | 0.324 | -4.267 | 1.266 | 1,30 | 0.269 | 0.205 | 0.396 | 1,30 | 0.534 |

**Table S3**. Mean ± standard error (ES) of the change (Δ) in body mass (g), bill colour (scale point), TAC (mmol Trolox equiv/l), GPx (nmol/min/mg protein), oxidative DNA damage (ng of 8-OHdG) and telomere length (T/S ratio) in all experimental groups.

|  | Constant diet | | | |  | Fluctuating diet | | | |
| --- | --- | --- | --- | --- | --- | --- | --- | --- | --- |
|  | Enlarged brood | | Reduced brood | |  | Enlarged brood | | Reduced brood | |
|  | Mean | SE | Mean | SE |  | Mean | SE | Mean | SE |
| Δ Body mass | -1.426 | 0.314 | -1.053 | 0.299 |  | -1.235 | 0.191 | -1.983 | 0.953 |
| Δ Bill colour | -1.230 | 0.460 | -1.424 | 0.363 |  | -3.020 | 0.418 | -1.527 | 0.335 |
| Δ TAC | 0.248 | 0.219 | 0.213 | 0.121 |  | 0.153 | 0.114 | -0.066 | 0.183 |
| Δ GPx | -221.621 | 111.650 | -624.811 | 284.458 |  | -1613.325 | 87.049 | -877.221 | 298.448 |
| Δ DNA damage | 3.782 | 1.059 | -0.488 | 0.465 |  | 2.906 | 1.049 | 1.137 | 0.949 |
| Δ Telomere length | -0.059 | 0.060 | 0.075 | 0.062 |  | -0.075 | 0.097 | -0.089 | 0.085 |

**Table S4**. Results of linear mixed models of the initial adult body mass, bill colouration, TAC level, GPx activity, and oxidative DNA damage level and telomere length. Variables retained in the final model are shown in bold. For removed terms, significance levels are those when terms were dropped from the model.

|  | Body mass | | | Bill colour | | | TAC | | | GPx | | | DNA damage | | | Telomere length | | |
| --- | --- | --- | --- | --- | --- | --- | --- | --- | --- | --- | --- | --- | --- | --- | --- | --- | --- | --- |
| *Source of variation* | F | d.f._n,d_ | p | F | d.f._n,d_ | p | F | d.f._n,d_ | P | F | d.f._n,d_ | p | F | d.f._n,d_ | p | F | d.f._n,d_ | p |
| Nutritional treatment | 0.02 | 1,34 | 0.900 | **6.65** | **1,35** | **0.014** | 0.29 | 1,36 | 0.590 | 0.51 | 1,36 | 0.480 | 0.01 | 1,34 | 0.908 | 1.20 | 1,36 | 0.280 |
| Brood size | 0.07 | 1,35 | 0.795 | 1.58 | 1,34 | 0.217 | 0.31 | 1,35 | 0.582 | 0.13 | 1,35 | 0.717 | 1.10 | 1,36 | 0.301 | 1.06 | 1,35 | 0.311 |
| Sex | 2.13 | 1,36 | 0.153 | **59.98** | **1,35** | **<0.001** | 0.10 | 1,34 | 0.752 | 0.18 | 1,34 | 0.676 | 0.85 | 1,35 | 0.361 | 0.29 | 1,34 | 0.595 |
| Nutritional treatment x brood size | 3.01 | 1,33 | 0.092 | 0.85 | 1,32 | 0.364 | 1.18 | 1,32 | 0.285 | 0.75 | 1,32 | 0.392 | 1.17 | 1,31 | 0.289 | 0.37 | 1,32 | 0.545 |
| Nutritional treatment x sex | 0.54 | 1,31 | 0.468 | 2.18 | 1,33 | 0.150 | 1.99 | 1,33 | 0.168 | 0.78 | 1,33 | 0.382 | 4.06 | 1,33 | 0.052 | 0.00 | 1,31 | 0.958 |
| Brood size x sex | 2.39 | 1,32 | 0.132 | 0.00 | 1,31 | 0.967 | 0.17 | 1,31 | 0.682 | 0.02 | 1,31 | 0.896 | 2.64 | 1,32 | 0.114 | 1.34 | 1,33 | 0.256 |
| Nutritional treatment x brood size x sex | 0.94 | 1,30 | 0.341 | 1.46 | 1,30 | 0.236 | 0.29 | 1,30 | 0.596 | 1.45 | 1,30 | 0.238 | 3.21 | 1,30 | 0.083 | 0.20 | 1,30 | 0.654 |
